# ^99m^Tc-labeled bevacizumab for detecting atherosclerotic plaque linked to plaque neovascularization and monitoring antiangiogenic effects of atorvastatin treatment in ApoE^−/−^ mice

**DOI:** 10.1038/s41598-017-03276-w

**Published:** 2017-06-14

**Authors:** Hui Tan, Jun Zhou, Xiangdong Yang, Mieradilijiang Abudupataer, Xiao Li, Yan Hu, Jie Xiao, Hongcheng Shi, Dengfeng Cheng

**Affiliations:** 1 Department of Nuclear Medicine, Zhongshan Hospital, Fudan University, Shanghai, 200032 China; 2 Institute of Nuclear Medicine, Fudan University, Shanghai, 200032 China; 3 Shanghai Institute of Medical Imaging, Shanghai, 200032 China; 4 Shanghai Institute of Cardiovascular Diseases, Zhongshan Hospital, Fudan University, Shanghai, 200032 China; 5 Department of Cardiac Surgery, Zhongshan Hospital, Fudan University, Shanghai, 200032 China

## Abstract

Atherosclerotic neovascularization plays a significant role in plaque instability as it provides additional lipids and inflammatory mediators to lesions, and resulting in intraplaque hemorrhage. Vascular endothelial growth factor-A (VEGF-A) is considered the predominant proangiogenic factor in angiogenesis. Bevacizumab, a humanized monoclonal antibody, specifically binds to all VEGF-A isoforms with high affinity. Therefore, in this study, we designed ^99m^Tc-MAG_3_-bevacizumab as a probe, and then investigated its usefulness as a new imaging agent for the detection of plaque neovessels, while also assessing the therapeutic effect of atorvastatin treatment. The ApoE^−/−^ mice treated with atorvastatin were used as the treatment group, and C57BL/6 J mice were selected as the control group. ^99m^Tc-MAG_3_-bevacizumab uptake was visualized on atherosclerotic lesions by non-invasive *in-vivo* micro-SPECT/CT and *ex-vivo* BSGI planar imaging. The value of P/B in each part of the aorta of ApoE^−/−^ mice was higher than in the treatment group and the C57BL/6 J mice, which was confirmed by Oil Red O staining, CD31 staining and VEGF immunohistochemistry staining. ^99m^Tc-MAG_3_-bevacizumab imaging allowed for the non-invasive diagnosis and assessment of plaque neovascularization. Furthermore, this probe may be used as a new molecular imaging agent to assess the antiangiogenic effect of atorvastatin.

## Introduction

Atherosclerotic cardiovascular diseases are still the leading causes of major morbidity and mortality in most countries around the world, most commonly triggered by vulnerable plaques resulting in acute cardiovascular events^1–3^. Therefore, the evaluation of atherosclerotic lesion instability plays a vital role in stratifying risk and providing early treatment. It is well known that intraplaque neovascularization is caused by an additional demand for oxygen and nutrients caused by the progression of atherosclerotic plaques. This progression is a considerable contributor to plaque destabilization and rupture because of the additional lipids and inflammatory mediators to lesions^4–6^. In addition, microvascular incompetence of neovascularization, which permits extravasation of erythrocytes into the plaque, is likely the source of intraplaque hemorrhage, further contributing to plaque rupture^7, 8^. Therefore, the development of accurate and feasible molecular imaging for assessing the presence of plaque neovascularization is crucial in recognizing active and vulnerable plaques.

Although a variety of factors have been found to contribute to the process of angiogenesis, vascular endothelial growth factor-A (VEGF-A) is recognized as the predominant proangiogenic factor^9, 10^. Prior studies have demonstrated that VEGF-A upregulation results in an increased permeability of vascular endothelial cell which caused immature microvessels^11, 12^. VEGF-A, the main proangiogenic isoform of the family, binds primarily to VEGF receptor (VEGFR)-1 and VEFGR-2^13^. Thus, molecular imaging of the components of VEGF-A or VEGFR should indicate the angiogenic process of plaques.

*In vivo* imaging of VEGFRs may be achieved by radiolabeled VEGF-A, but each different VEGF isoform has a different affinity for VEGFR-1 and VEGF-2, suggesting a potential role for VEGF-based imaging for neovascularization^14, 15^. Bevacizumab, a humanized monoclonal antibody, specifically binds to all VEGF-A isoforms with high affinity, and inhibits its interaction with VEGFR-1 and VEGFR-2, which is currently used in the clinic for cancer treatment and has been approved by the Food and Drug Administration (FDA)^16–19^. Several studies have used bevacizumab to target nuclear medicine probes by ^89^Zr, ^111^In and ^99m^Tc for labeling of tumor angiogenesis assessment^20–25^. To date, however, this probe of bevacizumab has not been used for plaque neovascularization *in vivo*. Golestani *et al*.^23^ used ^89^Zr- bevacizumab *ex vivo* imaging to evaluate excised carotid artery atherosclerotic plaque, and the results showed both that ^89^Zr-bevacizumab uptake was obviously correlated with VEGF immunohistochemical staining scores, and also that it is possible to detect VEGF using ^89^Zr-bevacizumab PET. Furthermore, both ^89^Zr and ^111^In are cyclotron produced with their own limitations. ^99m^Tc is the most widely used diagnostic radionuclide in nuclear medicine, as it has attractive nuclear properties and easy accessibility from a ^99^Mo/^99m^Tc generator system.

Therefore, in this study, we designed and prepared ^99m^Tc-labeled bevacizumab as a probe for plaque neovascularization imaging in an ApoE^−/−^ atherosclerotic mice model. In addition, previous studies have shown that treatment with stains for atherosclerosis (AS) can also exert antiangiogenic effects and reduce intraplaque neovascularization. Hence, we further investigated its usefulness as a new imaging agent to assess the therapeutic effect of atorvastatin, and to verify its antiangiogenic effects by *in vivo* molecular imaging.

## Results

### Probe preperation, stability, pharmacokinetics and biodistribution

**Figure 1:**
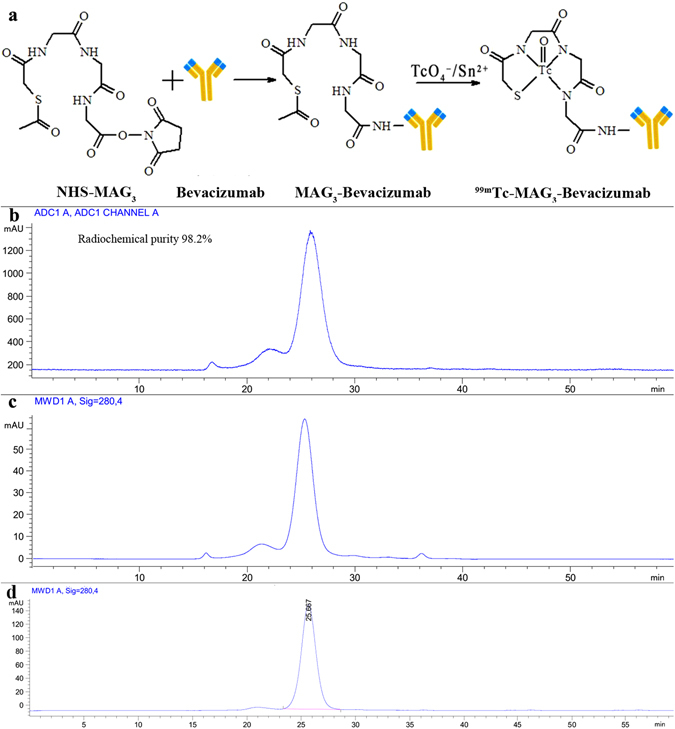
Synthetic procedures of ^99m^Tc-MAG_3_-bevacizumab and its quality control by size-exclusion HPLC. (**a**) The synthetic routes of ^99m^Tc-MAG_3_-bevacizumab. (**b**) The radio-HPLC analysis of ^99m^Tc-MAG_3_-bevacizumab. (**c**) The UV signal of MAG_3_-bevacizumab at 280 nm. (**d**) The UV signal of bevacizumab at 280 nm.

**Figure 2:**
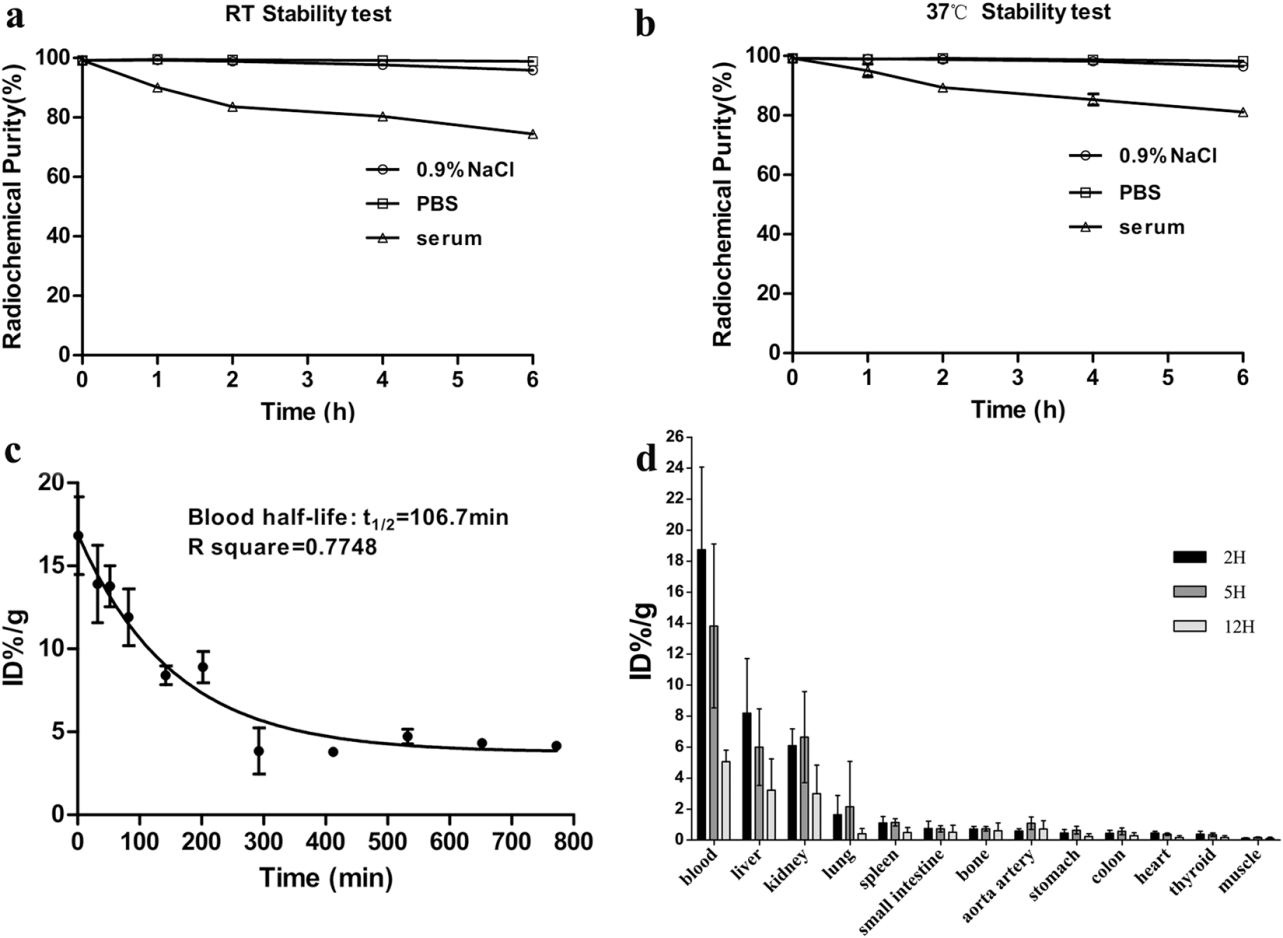
The stability, pharmacokinetics and biodistributions results of ^99m^Tc-MAG_3_-bevacizumab. (**a–b**) *In-vitro* stability test of the probe incubated in 0.9% NaCl, PBS and serum solution at room temperature or 37 °C. c, Pharmacokinetics of ^99m^Tc-MAG_3_-bevacizumab. e, Biodistribution results at 2 h, 5 h and 12 h post injection of the tracer. Abbreviation: PBS, phosphate buffered solution; %ID/g, percent injected dose per gram tissue.

The simple flowchart of ^99m^Tc-MAG_3_-bevacizumab synthesis is shown in Fig. 1a. The radiolabeling yield of ^99m^Tc-MAG_3_-bevacizumab was greater than 80%, and the radiochemical purity was 98.22%, as analyzed by radio-HPLC. The radioactivity peak overlapped with the ultraviolet (UV) peak of the probe (280 nm), and the retention time (*R*_*t*_) of the probe was 25.9 min, as shown in Fig. 1b,c and d. At both room temperature (RT) and at 37 °C, the probe showed favorable stability in 0.9% NaCl, PBS and serum at least 6 hours post labelling (Fig. 2a and b). ^99m^Tc-MAG_3_-bevacizumab was injected into wild-type C57BL/6 J mice to establish pharmacokinetics and biodistribution. The blood half-life of the probe was 106.7 min (R square = 0.7748; Fig. 1c). Biodistribution results at 2 hours, 5 hours and 12 hours p.i. were presented in Fig. 2d. The biodistribution results at 2 hours p.i. was as the following profile (%ID/g): blood, 18.74 ± 5.32; liver, 8.19 ± 3.54; kidneys, 6.09 ± 1.10; lung, 1.63 ± 1.24; spleen, 1.10 ± 0.43; small intestine, 0.76 ± 0.46; bone, 0.72 ± 0.16; aortic artery, 0.59 ± 0.14; stomach, 0.47 ± 0.23; colon, 0.45 ± 0.18; heart, 0.45 ± 0.12; thyroid, 0.38 ± 0.19; muscle, 0.12 ± 0.02. At 5 hours and 12 hours p.i., the biodistribution of ^99m^Tc-MAG_3_-bevacizumab in these organs tissues gradually decreased to the following profile comparing to 2 h p.i.: uptakes in aortic artery (1.09 ± 0.40 & 0.72 ± 0.53) and heart (0.37 ± 0.08 & 0.17 ± 0.10) were still low, but uptakes in blood (13.82 ± 5.29 & 5.07 ± 0.74), liver (6.00 ± 2.47 & 3.22 ± 2.02) and kidneys (6.64 ± 2.94 & 2.99 ± 1.85) were still high.

### Establishment of animal models and evaluation

**Figure 3:**
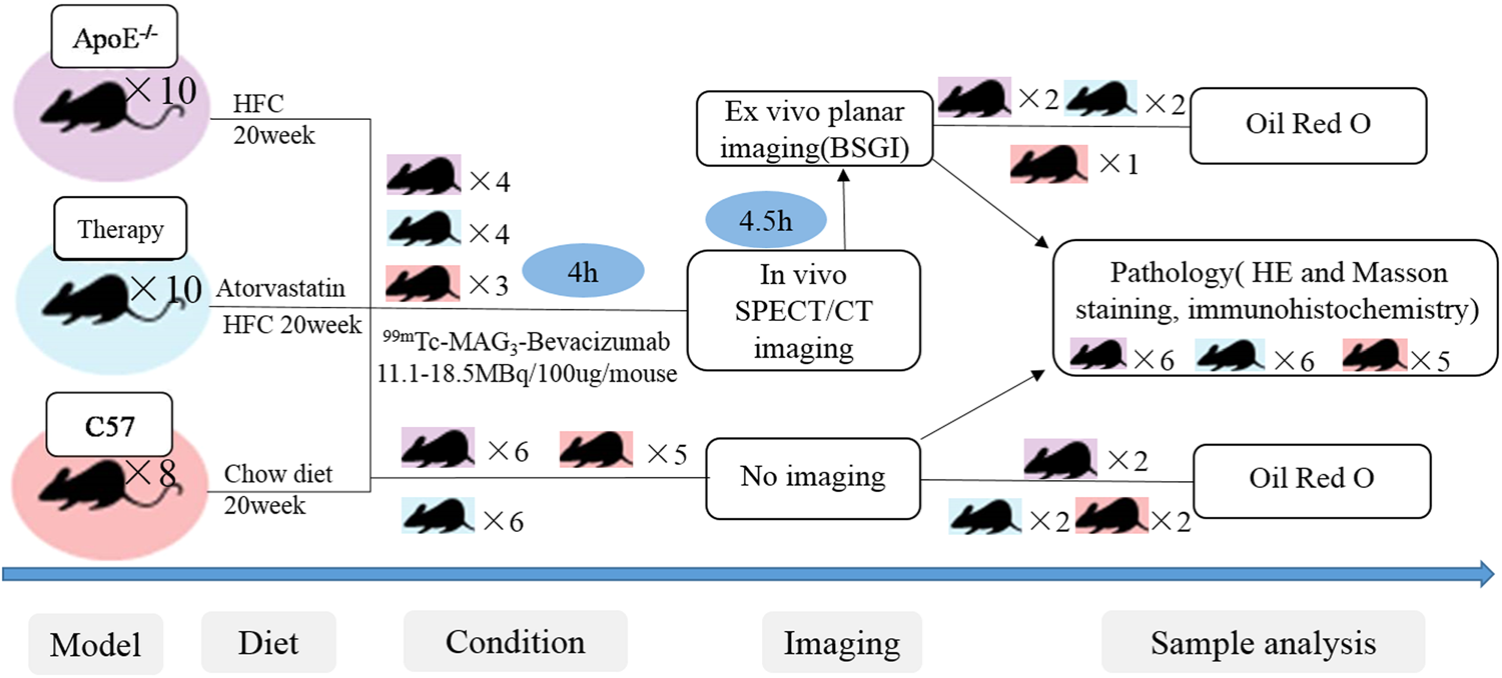
Flow chart of study design. Abbreviation: HFC, high fat/ high cholesterol; BSGI, breast specific gamma imaging.

**Figure 4:**
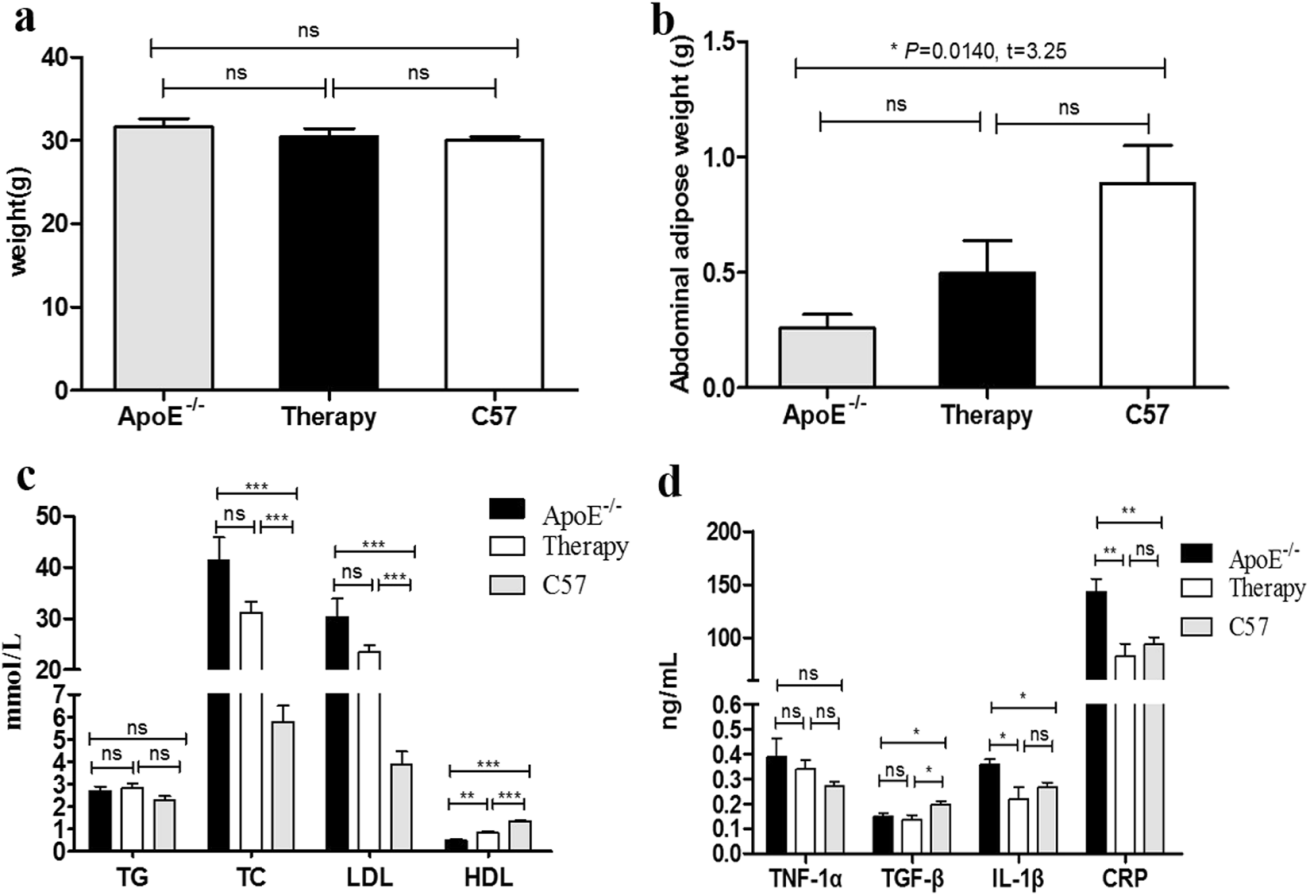
The weight, abdominal adipose weight, serological lipid profiles and cytokines of animal models. (**a–b**) Comparison of the body weight and the weight of abdominal adipose among the untreated, treated ApoE^−/−^ and C57BL/6 J mice. (**b–d**) Comparison of the level of serological lipid profiles and cytokines among the untreated, treated ApoE^−/−^ and C57BL/6 J mice. “*”, “**” and “***” indicated P < 0.05, < 0.01 and < 0.001, respectively; while “ns” denoted no significance.

The study design diagram is illustrated in Fig 3. The mean body weight of ApoE^−/−^ mice (31.62 ± 1.97 g) with a high fat/high cholesterol (HFC) diet was slightly heavier than C57BL/6 J mice (30.05 ± 0.88 g), as shown in Fig 4a. The weight of abdominal adipose tissue of ApoE^−/−^ mice was significantly less than C57BL/6 J mice (0.26 ± 0.12 g *vs*. 0.89 ± 0.37 g; *P = *0.0140, t = 3.25), but showed no difference between the ApoE^−/−^ and therapy groups or between the therapy and control groups (*P* > 0.05), which is shown in Fig. 4b.

The level of blood lipids is illustrated in Fig. 4c. The ApoE^−/−^ and therapy mice had higher concentrations of serological total cholesterol (TC) than did the C57BL/6 J mice (*P* < 0.0001, t = 7.64; *P* < 0.0001, t = 11.72), but the same level of low-density lipoproteins (LDL) (*P* < 0.0001, t = 7.27; *P* < 0.0001, t = 13.93). The levels of TC or LDL in the serum of atorvastatin-treated ApoE^−/−^ mice showed slight reduction compared to ApoE^−/−^ mice, though they failed to reach a statistical significance (*P* > 0.05). Compared to the groups of ApoE^−/−^ and C57BL/6 J, therapy and C57BL/6 J, and ApoE^−/−^ and therapy, mice showed lower concentrations of serological high-density lipoprotein (HDL), (P < 0.0001, t = 12.95; P < 0.0001, t = 8.13; P = 0.0011, t = 4.94). While inter-group results were not significantly different (*P* > 0.05), there was also no obvious significance between serological triglyceride (TG) levels of the ApoE^−/−^, therapy and C57BL/6 J group (*P* > 0.05).

Furthermore, the levels of serological interleukin-1β (IL-1β) and C-reactive protein (CRP) were noted to be higher in ApoE^−/−^ mice than in C57BL/6 J mice (*P* = 0.0137, t = 3.27; *P* = 0.0070, t = 3.76) and treatment mice (*P* = 0.0467, t = 2.41; *P* = 0.0093, t = 3.56). However, transforming growth factor β (TGF-β) was significantly lower in the ApoE^−/−^ mice (*P* = 0.0311, t = 2.69) and the therapy mice (*P* = 0.0262, t = 2.71) than in the C57BL/6 J mice (Fig. 4d). The inter-group differences of serological tumor necrosis factor-1α (TNF-1α) were not significant (*P* > 0.05) (Fig. 4d).

### Immunoreactivity of ^99m^Tc-MAG_3_-bevacizumab

^99m^Tc-MAG_3_-bevacizumab was shown 26.36% ± 6.13% binding to VEGF165 coated plates. Moreover, a competition assay with excess unlabeled bevacizumab (1000 fold) displayed completed blocking of ^99m^Tc-MAG_3_-bevacizumab to VEGF165 with only 2.63% ± 0.47% binding.

### *In vivo* Micro-SPECT/CT and *ex vivo* BSGI imaging

**Figure 5:**
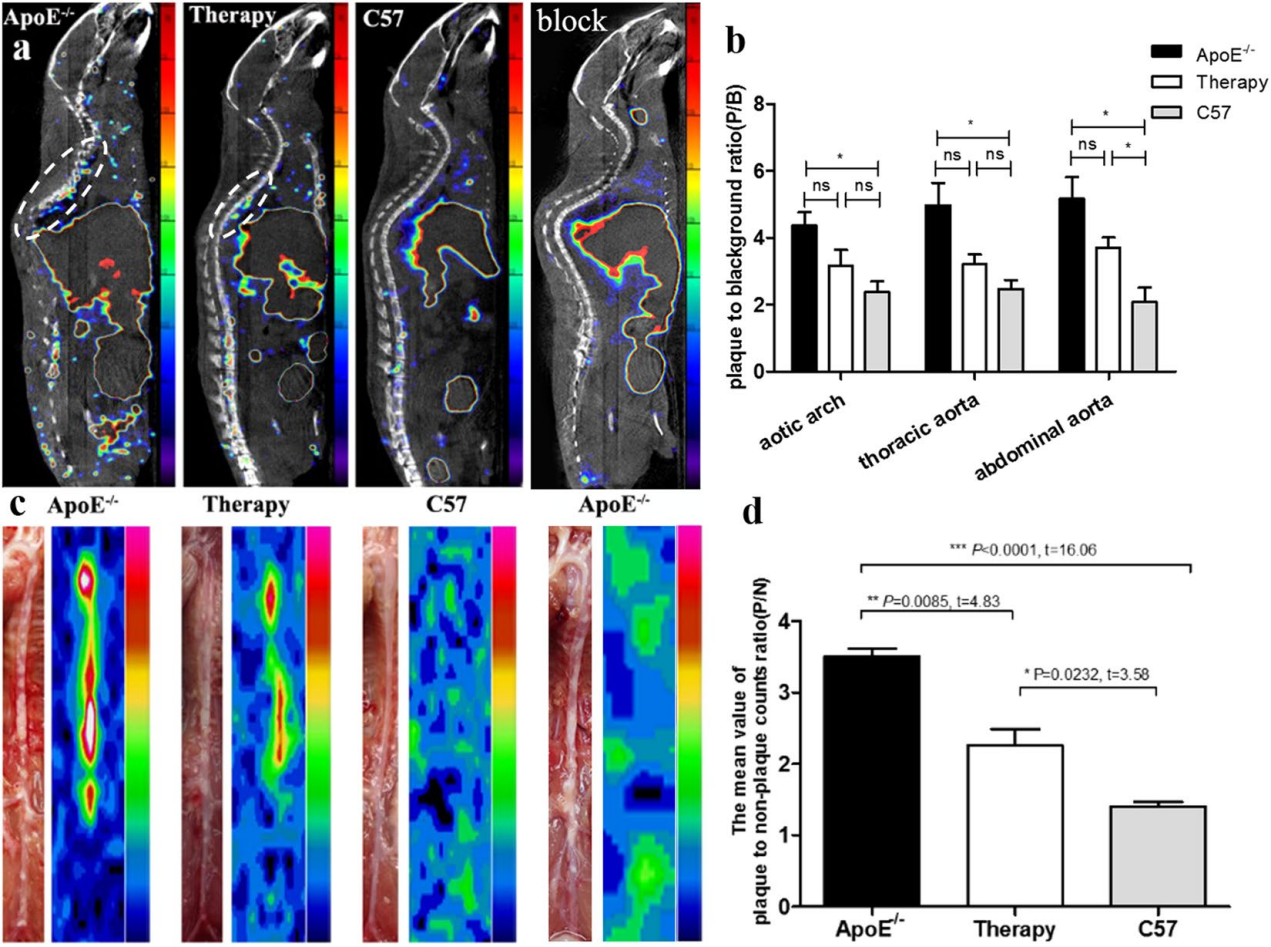
*In-vivo* micro-SPECT/CT, *ex-vivo* BSGI imaging and semi-quantitative analysis. (**a**) Micro-SPECT/CT images (saggital) of untreated, treated ApoE^−/−^, C57BL/6 J mice and ApoE^−/−^ mice with blocking at 4 h post injection of ^99m^Tc-MAG_3_-bevacizumab, tracer accumulated area in thoracic aorta highlighted with oval white dotted line. (**b**) The semi-quantitative analysis results of micro-SPECT/CT scannings expressed as plaque-to-background ratio (P/B) for different parts of aortic plaque radioactivity uptake among different groups. (**c**) *Ex vivo* planar imaging of aorta by breast specific gamma imaging (BSGI). (**d**) The semi-quantitative analysis of mean value of plaque to non-plaque counts ratio (P/N) for BSGI imaging among untreated, treated ApoE^−/−^ and C57BL/6 J mice groups.

*In vivo* micro SPECT/CT imaging, ^99m^Tc-MAG_3_-bevacizumab uptake allowed the noninvasive visualization of atherosclerotic plaques, and the tracer uptake was higher in the ApoE^−/−^ mouse group than in the treatment and C57BL/6 J groups by visual analysis (Fig. 5a). In the ApoE^−/−^ mouse blocked with 1000 μg bevacizumab, no radioactivity uptake was found in aorta (Fig. 5a), and similar result was displayed in *ex vivo* BSGI imaging (Fig. 5c). In addition, the results of a semi-quantitative analysis of SPECT/CT, expressed as plaque to background ratio (P/B), also showed that ApoE^−/−^ mice had a significantly higher level of radioactivity than the C57BL/6 J group at the aortic arch (P/B: 4.36 ± 0.72 versus 2.38 ± 0.56, *P* = 0.0198, t = 2.72), the thoracic aorta (P/B: 4.97 ± 1.16 versus 2.48 ± 0.47, *P* = 0.0261, t = 3.45) and the abdominal aorta (P/B: 5.17 ± 1.11 versus 2.07 ± 0.76, *P* = 0.0165, t = 3.97), as seen in Fig. 5b. The treatment group showed a lower tracer uptake rate than ApoE^−/−^ mice at the aortic arch (P/B: 3.17 ± 0.81), the thoracic aorta (P/B: 3.21 ± 0.51) and the abdominal aorta (P/B: 3.70 ± 0.54). However, there was no significant difference (*P* > 0.05) (Fig. 5b). Compared with C57BL/6 J mice, the mean value of P/B in the treatment group was higher with statistical significance (*P* = 0.0384, t = 3.04) only at the abdominal aorta (Fig. 5b).

After SPECT/CT imaging, we immediately took out the aorta and performed *ex vivo* planar imaging by breast specific gamma imaging (BSGI) for the excised aortas. Figure 5c illustrates the gross morphology of the aortic plaque, which confirms the SPECT/CT and corresponding BSGI imaging finding that the more plaque in an area of the aorta, the more tracer uptake in that area. In addition, ^99m^Tc-MAG_3_-bevacizumab uptake in ApoE^−/−^ mice was higher than in the treatment and control groups. The therapeutic group was also higher than the C57BL/6 J group by visual analysis. And the same semi-quantitative analysis of mean value of plaque to non-plaque counts ratio (P/N), ApoE^−/−^ versus treated group (P/N: 3.50 ± 0.20 versus 2.26 ± 0.40, *P* = 0.0085, t = 4.83), ApoE^−/−^ versus C57BL/6 J group (P/N: 3.50 ± 0.20 versus 1.40 ± 0.11, *P* < 0.0001, t = 16.06), treated versus C57BL/6 J group (*P* = 0.0232, t = 3.58), shown in Fig. 5d.

### Oil Red O staining

**Figure 6:**
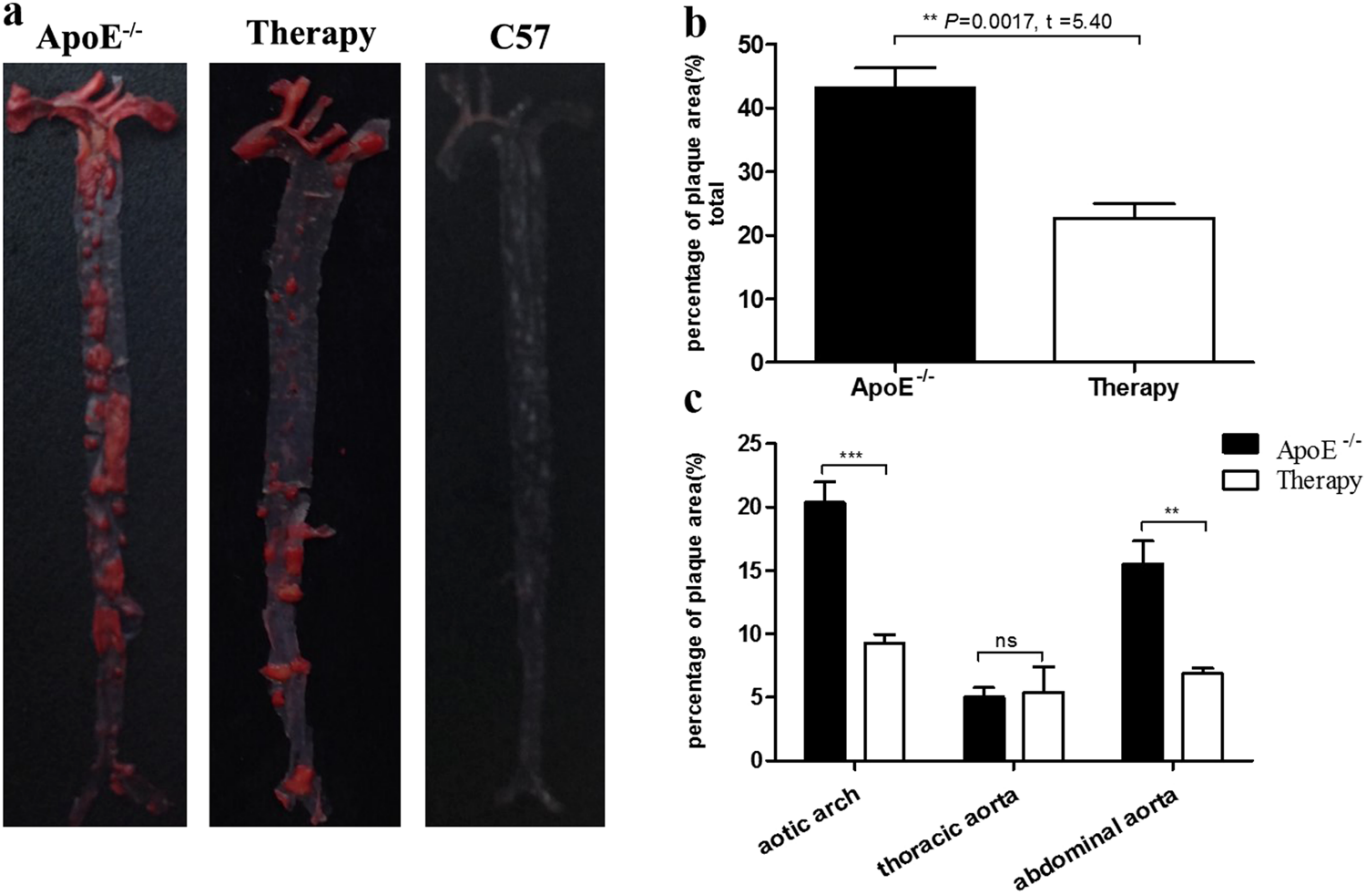
Oil Red O staining. (**a**) Oil Red O staining of untreated, treated ApoE^−/−^ and C57BL/6 J mice. (**b**) Percentage of Oil Red O stained area analysis. (**c**) Comparison of lipid plaque by Oil Red O staining in aortic arch, thoracic and abdominal parts of aorta.

Next, Oil Red O staining was performed on all mice groups to compare the percentage of plaque areas in both the total aorta and specific parts of the aorta, as shown in Fig. 6. The ApoE^−/−^ mice presented with a significantly higher percentage area of aortic lipid plaque than did the treated mice (43.24 ± 6.13% *vs*. 22.67 ± 4.53%; *P = *0.0017, t = 5.40), as shown in Fig. 6a and b. There was no typical plaque was observed in the aorta of C57BL/6 J mice. Furthermore, the percentage area of plaque in the aortic arch and abdominal aorta in ApoE^−/−^ mice were both higher than treated mice, with statistical significance (*P = *0.0007, t = 6.37) and (*P = *0.0037, t = 4.60). The difference in percentage area of plaque in the thoracic area, however, was not statistically significant (*P* > 0.05) (Fig. 6c) between the groups.

### HE and Masson trichrome staining, CD31 and VEGF immunohistochemistry

**Figure 7:**
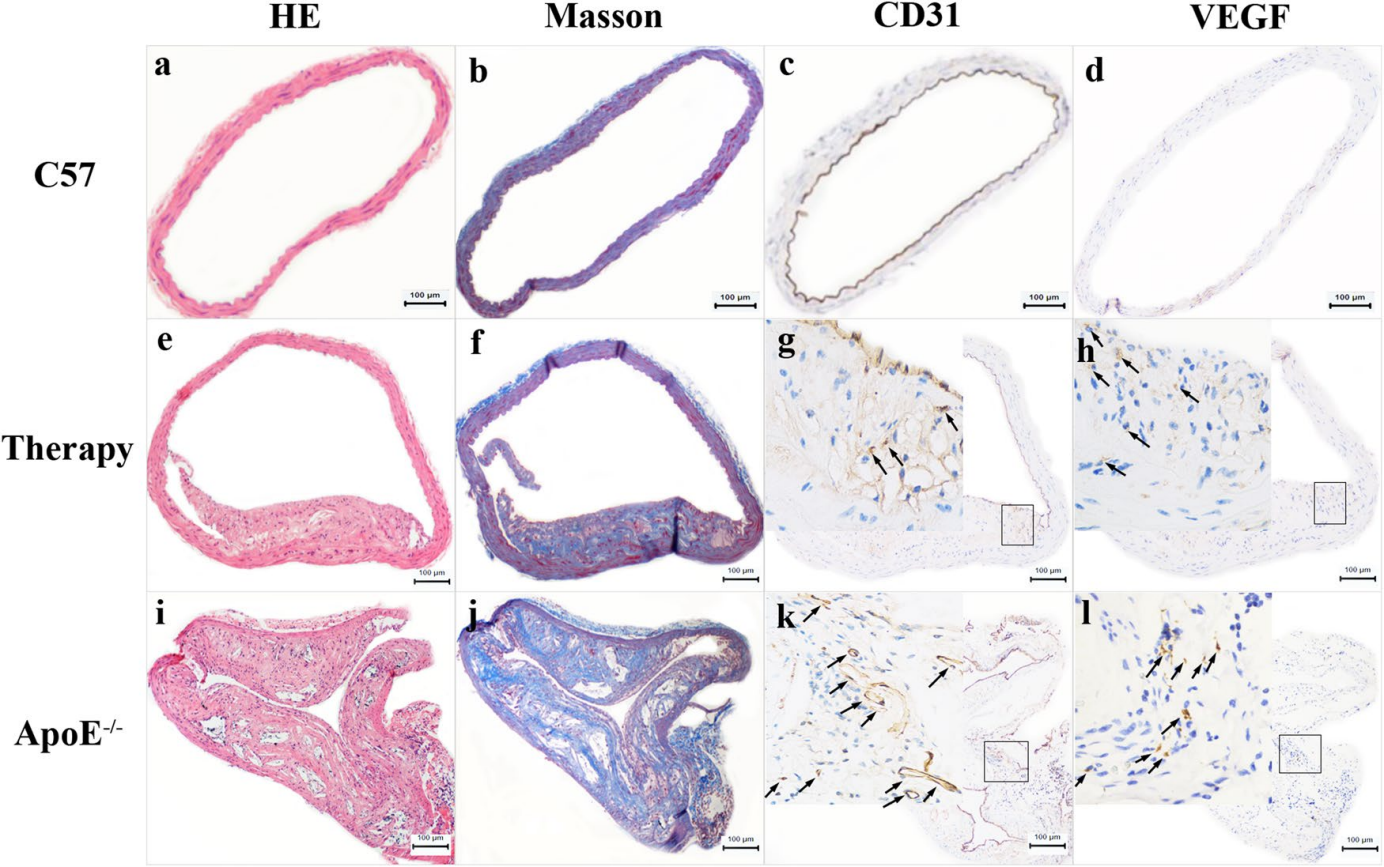
Pathological results. (**a**–**d**) The pathological results of C57BL/6 J mice: HE staining (×100), Masson staining (×100), CD31(×100) and VEGF (×100) immunohistochemical staining displayed from left to right in turn. (**e**–**h**) The pathological results of treated mice: HE staining (×100), Masson staining (×100), CD31(×100 & ×400) and VEGF (×100 & ×400) immunohistochemical staining displayed from left to right in turn. (**i**–**l**) The pathological results of ApoE^−/−^ mice: HE staining (×100), Masson staining (×100), CD31(×100 & ×400) and VEGF (×100 & ×400) immunohistochemical staining displayed from left to right in turn.

As shown in Fig. 7a–d, there was no plaque in the wild-type C57BL/6 J mice by HE staining, Masson trichrome staining, or CD31 and VEGF immunohistochemistry. Compared to the ApoE^−/−^ mice, the group treated with atorvastatin exhibited a reduction in atherosclerotic plaque and stenosis ratio of the aorta, and the numbers of new vessels in plaques were markedly decreased, as displayed in Fig. 7e–h. While the ApoE^−/−^ mice had an aorta with obvious plaque as identified by HE staining, the Masson staining only displayed intraplaque collagen (Fig. 7i,j). In addition, the images of CD31 and VEGF immunohistochemistry showed intraplaque with neovascularization (Fig. 7k,l).

### The aorta *in-vivo* SPECT/CT imaging and pathology

**Figure 8:**
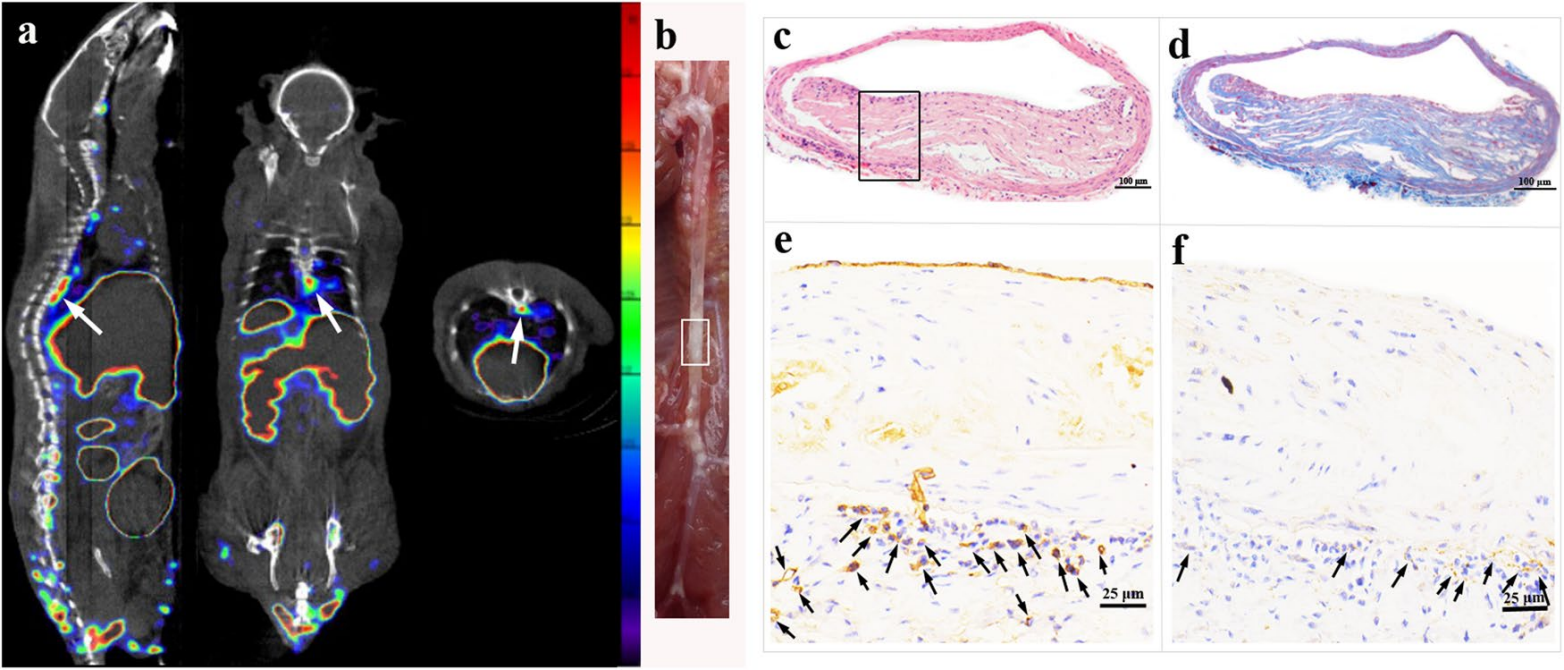
*In-vivo* SPECT/CT imaging and pathological results. (**a**) *In vivo* micro-SPECT/CT images of ^99m^Tc-MAG_3_-bevacizumab (sagittal, coronal, and transverse views, left to right) in an ApoE^−/−^ mouse. (**b**) The gross morphology of aortic plaque. (**c**) IHC results of slices from intense radioactivity accumulated plaque area in aortic arch, CD31 immunohistochemical image (×100), higher magnification (×400), VEGF immunohistochemical image (×100) and higher magnification (×400) displayed from left to right in turn. (**d**) IHC results of slices from intense radioactivity accumulated plaque area in thoracic aorta, CD31 immunohistochemical image (×100), higher magnification (×400), VEGF immunohistochemical image (×100) and higher magnification (×400) displayed from left to right in turn.

HE and Masson trichrome staining and CD31 and VEGF immunohistochemistry staining were performed on the aorta sections sliced at points with obvious radioactivity in SPECT/CT images. The lesions with accumulated radioactivity were consistent with the anatomical structure of the plaques. Interestingly, the thoracic aorta with the most radioactivity showed the most distinct plaque in corresponding areas (Fig. 8a,b). Figure 8e and f further illustrate that the level of ^99m^Tc-MAG_3_-bevacizumab uptake in plaque was primarily related to the numbers of new blood vessels in the plaque on pathological results.

## Discussion

To our current knowledge, plaque neovascularization is recognized as a critical process of the progression of atherosclerotic plaques and is related to plaque vulnerability. Histopathological analysis has been the gold standard for visualizing newly formed vessels in unstable lesions, though it has the disadvantage of requiring tissue samples. Functional or molecular imaging techniques, however, have demonstrated distinct visualization of plaque microvessels non-invasively. Bevacizumab, a humanized monoclonal antibody against VEGF-A with reduced immunogenicity, is currently approved by the FDA to treat some cancers, inhibiting tumor angiogenesis in the clinic setting. However, it also shows serious adverse effects, including cardiovascular diseases, such as hypertension and thromboembolism, especially for the cancer patient with pre-existing arterial cardiovascular disease^26–28^. For that reason, screening for tumor patients with a high risk for atherothrombotic disease is crucial to avoid the serious complications of bevacizumab treatment. Bevacizumab radionuclide imaging, as a noninvasive method, not only could be quite helpful in guiding bevacizumab therapy for many cancers because of interpatient tumor heterogeneity in tumor imaging aspect, maybe but may also discourage some tumor patients with unstable plaque from receiving bevacizumab therapy to avoid the serious complications in atherosclerotic imaging aspect. Therefore, we applied ^99m^Tc-MAG_3_-bevacizumab as a probe for atherosclerotic imaging to reflect microvessels of plaque linked to plaque instability in this study.

The study presented an indirect method for ^99m^Tc labeling bevacizumab, applied NHS-MAG_3_ as a bifunctional chelator, which yielded good radiolabeling yields that reached over 80%, similar to ^99m^Tc carbonyl DTPA-bevacizumab reported by the Kameswaran M, *et al*.^25^. The immunoreactivity results confirmed that bevacizumab still kept its reactivity to the VEGF receptor after labeling with ^99m^Tc. The biodistribution pattern of ^99m^Tc-MAG_3_-bevacizumab in wild-type C57BL/6 J mice indicated a high radioactivity in the blood over the times observed, which could be because of bevacizumab with a relatively long biological half-life of approximately 3 weeks. The fact that the probe was eliminated through both the kidneys and the hepatobiliary system may be a reason for the high accumulations of radioactivity in those organs. The high radioactivity in these organs is consistent with previous studies that showed biodistribution of both probes ^99m^Tc(CO)_3_-bevacizumab and ^99m^Tc-carbonyl-DTPA-bevacizumab^25, 29^. The aortic artery and heart with relatively low ^99m^Tc-MAG_3_-bevacizumab uptake may illustrate the potential value for AS imaging. Unfortunately, the probe showed relatively slow clearance rates in pharmacokinetic experiments, and this rate will have resulted in a low signal to noise ratio (SNR) for diagnostic imaging. These findings are similar to those in previous studies that fabricated these probes of ^99m^Tc-carbonyl-DTPA-bevacizumab, ^124^I-iodinated-VG76e and ^123^I-VEGF for imaging VEGF *in vivo* ^25, 30, 31^. Thus, new molecular probes need to address this shortcoming in our study later.

In our experiments, ApoE^−/−^, treated mice and C57BL/6 J models were established, and the level of serum lipid analyses revealed obvious higher in ApoE^−/−^ than in C57BL/6 J mice in TC and LDL, but treated group without markedly decreasing than ApoE^−/−^ mice, just as the previous studies showed that 10 mg/kg/d atorvastatin treatment did not lower plasma total cholesterol levels^32–34^. And the protective index of serum HDL showed remarkably difference among intergroup. The classical pro-inflammatory cytokines (IL-1β and CRP) were significantly increased in ApoE^−/−^ mice, compared to C57BL/6 J mice, especially the IL-1β, which played a central role in regulating immune, and inflammation response. Compared with ApoE^−/−^ mice, the treated group with 10 mg/kg/d atorvastatin displayed the decreased serum IL-1β and CRP levels. So, our study demonstrated the beneficial effect of atorvastatin is downregulated inflammatory cytokines with the inhibition of macrophage infiltration, in accord with the previous reach performed by Nie P, *et al*.^32^. The role of TGF-β in atherosclerotic progression is controversial, and studies largely supported an anti-atherogenic role of TGF-β^34^, with revealing a negative correlation between plasma TGF-β level and the extent of AS, then our study also confirmed this conclusion that the level of plasma TGF-β was significantly lower in ApoE^−/−^ mice and therapy mice than C57BL/6 J mice.

There were few studies used bevacizumab as a probe to image plaque neovasculature, just Golestani *et al*.^23^ displayed the results that ^89^Zr-bevacizumab uptake in the excised carotid artery atherosclerotic plaques for *ex vivo* imaging, was obvious correlated with VEGF immunohistochemical staining scores. In our study, the results showed that *in vivo* ^99m^Tc-MAG_3_-bevacizumab micro SPECT/CT imaging was allowed noninvasive visualization of atherosclerotic plaques, and the tracer uptake was higher in ApoE^−/−^ than C57BL/6 J group by visual and semi-quantitative analysis. The results of *ex-vivo* planar view was just similar to SPECT/CT imaging that obvious radioactivity uptake in ApoE^−/−^ not in C57BL/6 J group both visual and semi-quantitative analysis, and was direct reflected the tracer uptake in atherosclerotic plaques because of it avoiding interference from the surrounding tissue with high radioactivity accumulation. ^99m^Tc-MAG_3_-bevacizumab concentration in aortic plaques *in-vivo* and *ex-vivo* imaging was further confirmed by gross morphology, Oil Red O staining, CD31 and VEGF immunohistochemistry staining of plaque. Oil Red O staining was used to assess lipid deposition with arterial lumina^35^. As for the immunohistochemistry, it was the gold standard to look into neovascularization in plaques. Our study indicates that ^99m^Tc-MAG3-bevacizumab could be an available probe for atherosclerotic angiogenesis imaging, non-invasively, and evaluate neovascularization of plaques that tightly linked to plaque rupture.

It is well known that atorvastatin as HMG-CoA reductase is broadly applied in lipid-lowering treatment, and some research had shown it also with pleiotropic effects, which included anti-angiogenic effects^32, 33, 36, 37^. Roth *et al*.^36^ reported that atorvastatin treatment was responsible for reduction in the expression of inflammatory cytokines, coronary stenosis, and the occurrence of intraplque microvessels. Our results also had proved atorvastatin therapy with pleiotropic effects in decreasing the level of plasma inflammatory factors, plaques area of aorta and stenosis of arterial lumen which confirmed by Oil Red O staining, HE and Masson staining, as well as atherosclerotic neovascularization which affirmed by immunohistochemistry.

It has been presented in previous studies that molecular imaging with targeted MRI, nuclear medicine and ultrasound probes can be applied for assessing the effect of stains treatment in AS, but most were to assess the anti-inflammatory treatment of stains^38–40^. In this study, ^99m^Tc-MAG3-bevacizumab as SPECT probes were used for assessing the anti-angiogenic effects for atorvastatin therapy in AS model mice. Our results showed that the treatment group with ^99m^Tc-MAG_3_-bevacizumab uptake was lower than that of ApoE^−/−^ mice in *in-vivo* SPECT/CT imaging by visual analysis, and *ex-vivo* BSGI imaging both visual and semi-quantitative analysis, and these imaging results were consistent with pathological analysis. This study revealed the potential of ^99m^Tc-MAG_3_-bevacizumab as a molecular imaging probe to non-invasively assess the effects of antiangiogenic.

In summary, this study demonstrated that a ^99m^Tc-MAG_3_-bevacizumab targeted SPECT probe allowed for non-invasively diagnosing and assessing the neoangiogenesis of atherosclerotic plaques in ApoE^−/−^ mice. It is a potential probe to not only guide bevacizumab therapy because of interpatient heterogeneity, but also to screen suitable patients with bevacizumab treatment to avoid serious complications in cardiovascular diseases. Furthermore, this probe as a new molecular imaging agent could be used to assess the antiangiogenic effect of atorvastatin.

## Methods

### Approvals

The experimental protocols were approved by the medical ethics of committee Zhongshan Hospital, Fudan University.

### Design and Preparation of ^99m^Tc-MAG_3_-bevacizumab

Bevacizumab (Roche Pharma Ltd., Reinach, Switzerland), a humanized monoclonal antibody with 149 kDa against VEGF-A was selected as a targeting molecule. N-hydroxysuccinimidyl S-acetylmercaptoacetyl -triglycinate (NHS-MAG_3_) (WuXi APPTec, Shanghai, China) was conjugated to bevacizumab as a bifunctional chelator following the established protocol^41, 42^. ^99m^Tc-pertechnetate (^99m^TcO_4_^−^) (GMS Pharmaceutical Co., Ltd., Shanghai, China) was eluted in saline solution on a daily basis.

The process of ^99m^Tc-MAG_3_-bevacizumab synthesis was as follows: we added 100 μg bevacizumab in 0.30 M HEPES to a tube containing NHS-MAG_3_ powder, and then incubated the mixture at room temperature for 1 hour with occasional agitation. The mixture was purified by the centrifugation method to remove unconjugated and hydrolyzed MAG_3_ using 0.25 M ammonium acetate as eluent, to obtain MAG_3_-bevacizumab. Then, MAG_3_-bevacizumab conjugate was added to a combined solution of 45 μL ammonium acetate (0.25 M) and 15 μL tartrate buffer, with no more than 25 μL (around 37MBq) ^99m^TcO_4_^−^. Immediately, 4 μL of freshly prepared 1 mg/mL SnCl_2_∙2H_2_O solution were added. After gently vortexing for a short time, the combined solution was incubated at room temperature for 1 hour. The purification of ^99m^Tc-MAG_3_-bevacizumab was by centrifugation, and its radiochemical purity was measured by a size-exclusion HPLC shift assay (Agilent Technologies, 1260 Infinity).

### *In vitro* stability, pharmacokinetics and biodistribution of ^99m^Tc-MAG_3_-bevacizumab

The *in vitro* stability in 0.9% NaCl, 0.05 M PBS and serum was evaluated at 1, 2, 4, and 6 h at room temperature and 37 °C. Male wild-type C57BL/6 J (n = 20) mice with the age of 8 weeks were injected via the caudal vein with ^99m^Tc-MAG_3_-bevacizumab at a dose of 10 μg/0.74MBq/mouse. For pharmacokinetics study (n = 5), five microliters of blood from the tail vein were collected at serial time points. For a biodistribution study (n = 15), 5 mice per group were anesthetized, then killed at 2 hours, 5 hours and 12 hours post injection. Tissues from blood, liver, kidney, lung, spleen, small intestine, bone, aorta artery, stomach, colon, heart, thyroid, and muscle were harvested. Each sample was weighed and counts were recorded with a counter (CRC-15R, Capintec Inc., Ramsey, NJ). After corrected for radioactive decay, data were calculated as a percent of injected dose per gram of tissue (ID%/g). The mean blood half-life of ^99m^Tc-MAG_3_-bevacizumab was analyzed by monoexponential decay using GraphPad Prism 5.01 (GraphPad Software Inc.).

### Immunoreactivity of ^99m^Tc-MAG_3_-bevacizumab

The immunoreactivity assay was conducted according to previous reported^43, 44^. Each well in ELISA plates (Corning Incorporated, USA) was coated with 5 μg/ml of recombinant human VEGF165 (R&D Systems) at 4 °C overnight, and then VEGF165 solution was adjusted to pH 9.6 by adding 15 mM Na_2_CO_3_ and 35 mM NaHCO_3_. Thereafter, the wells were blocked with 100 μL 1% human serum albumin (HSA, Sigma) in 0.067 M PBS. After blocking, the wells were washed three times with 0.1% polysorbate 80 (Sigma) in 0.067 M PBS. For the following binding assay, 10 ng/ml of ^99m^Tc-MAG_3_-bevacizumab was added to the wells with VEGF coated. All wells were allowed to incubate for 2 h at room temperature. Subsequently, unbound antibody was removed by washing the wells three times with 0.1% polysorbate 80 in 0.067 M PBS. Finally, the bound antibody was solubilized with 0.2 M NaOH and withdrawn for gamma counting. The percentage of binding was calculated as bound counts divided by total counts. Competition experiments were performed by adding an excess of unlabeled bevacizumab (1000 fold) to the wells one hour in advance before ^99m^Tc-MAG_3_-bevacizumab was added into VEGF165 coated wells. Each group was conducted in triplicate.

### Animal protocol

Wild-type C57BL/6 J (n = 28) and ApoE^−/−^ mice (n = 20) (male, 8weeks) were purchased from Cavens Laboratory Animal Center (Changzhou, China). C57BL/6 J mice with the age of 8 weeks were used for pharmacokinetics study (n = 5) and biodistribution study (n = 15). The rest C57BL/6 J mice (n = 8) were fed standard chow containing 4% to 6% fat and a cholesterol content <0.02% for 20 weeks and served as controls for imaging and pathology, showed in Fig. 3. The ApoE^−/−^ mice (n = 10) fed with a Western diet (containing 21% fat and 0.15% cholesterol by weight) with a high fat/high cholesterol (HFC) diet for 20 weeks without atorvastatin therapy were used for imaging and pathology, displayed in Fig. 3. The ApoE^−/−^ mice as treatment group (n = 10) were given 12 weeks of atorvastatin therapy with 10 mg/kg/d after 8 weeks on a Western diet applied for imaging and pathology (Fig. 3). All mice were kept in a temperature-controlled environment with a 12-hour light/dark cycle with free access to food and water. Animal care and experimental procedures were conducted in compliance with the Helsinki Declaration, and were approved by an institutional animal care committee.

### The basic information of mice model and its serological biochemical analysis

All mice, both with and without imaging, fasted for 4 hours. Body weight and the weight of abdominal fat were measured after general anesthesia by intraperitoneal injection of 4% chloral hydrate (400 mg/kg). In addition, blood samples were collected by left-ventricle puncture. After coagulating for 20 min at room temperature, the blood was centrifuged (3000 rpm × 10 min) to extract supernatant serum. Plasma TG, TC, HDL, and LDL levels were then measured enzymatically with commercial kits. Enzyme-linked immunosorbent assay (Elisa) kits were used to test serological TNF-1α, TGF-β, IL-1β and CRP. Assays were performed according to manufacturer’s protocols, using a microplate reader for Elisa (Denley Dragon Wellscan MK 3, Thermo, Finland).

### *In vivo* micro-SPECT/CT imaging and semi-quantitative analysis

After the feeding at 20 weeks, ^99m^Tc-MAG_3_-bevacizumab (11.1-18.5MBq/100 μg/mouse) was injected to the caudal vein of mice in the ApoE^−/−^ group (n = 4), the treatment group (n = 4) and the C57BL/6 J mice as a control group (n = 3). For blocking study, 1000 μg bevacizumab was pre-injected to the caudal vein of ApoE^−/−^ mice (n = 2), 1 h later, ^99m^Tc-MAG_3_-bevacizumab (18.5MBq/100 μg/mouse) was injected by the caudal vein. Four hours after this injection, mice were anesthetized with 2% isoflurane inhalation, and *in-vivo* micro-single photon emission computed tomography/computed tomography (SPECT/CT) imaging was performed on a Nano SPECT/CT scanner (Bioscan, Washington DC, USA). The CT was performed first with the following parameters: frame resolution, 256 × 512; tube voltage, 45 kVp; current, 0.15 mA; and exposure time, 500 ms/frame. SPECT was then performed in same bed position as the CT with the following parameters: four high-resolution conical collimators with 9-pinhole plates; energy peak, 140 keV; window width, 10%; resolution, 1 mm/pixel; matrix, 256 × 256; and scan time, 30 s/frame. 3-dimensional ordered subset expectation maximization images were reconstructed using a HiSPECT algorithm.

Micro-SPECT/CT data were transferred to software *InVivo* Scope (Version 1.43, Bioscan, Washington DC, USA) for processing. For each small-animal SPECT scan, 3-dimensional regions of interest (ROI) were drawn in the aortic arch, thoracic aorta and abdominal aorta in areas of obvious radioactivity (Fig. 6A). Another ROI of corresponding size was drawn in an area of surrounding waist muscle in comparison as background activity. The concentration of radioactivity (mCi/mm^3^) was automatically generated by the software for each ROI. ^99m^Tc-MAG_3_-bevacizumab uptake in each section of aortic plaque was expressed as a ratio for each ROI of plaque signal to background signal (P/B), as the semi-quantitative analysis index referenced in our previous research method^45^.

### *Ex vivo* BSGI imaging and semi-quantitative analysis

After micro-SPECT/CT imaging, the aortas were immediately removed for *ex-vivo* planar imaging by BSGI to eliminate adjacent tissue interference and to further verify signal intensity among different mice. BSGI was performed with the following parameters: collimator, low-energy general-purpose; peak energy, 140 keV; window width, 10%; resolution, 0.32 mm/pixel; matrix, 80 × 80. The collection time was 30 min with total counts of 80000–100000. The semi-quantitative analysis of ^99m^Tc-MAG_3_-bevacizumab uptake in BSGI of aortic plaque was expressed as the value of plaque to non-plaque counts ratio (P/N)^46^.

### Oil-Red-O staining of aorta and analysis

After blood was drawn from each mouse, the circulation system was washed with saline, and then fixed with a 4% paraformaldehyde twice. The aorta was excised after the removal of aortic peripheral adipose tissue, and then was cut longitudinally to expose the intimal surface. At this time, samples were fixed in 4% paraformaldehyde for 24 hours. Two aortas of ApoE^−/−^ mice, treatment mice and C57BL/6 J mice that experienced *in-vivo* SPECT/CT imaging and BSGI were selected for Oil-Red-O staining, as were two aortas of ApoE^−/−^ and treatment mice and one control mouse without imaging (Fig. 3). The staining method is as follows: the fixed aortas were rinsed with 60% propylene glycol for 5 min and stained in 0.5% Oil-Red-O solution (Sigma) for 45 min. The aortas were then differentiated in a 60% propylene glycol solution for 5 min. The stained aortas were spread on a black charpie for photographing using a digital camera under identical light conditions with same the photographing parameters. Pictures were analyzed with Image Pro Plus (Version 6.0, Media Cybernetics, Washington DC, USA).

### Histological Evaluation

Two aortas of ApoE^−/−^ mice, treatment mice and C57BL/6 J mice that underwent *in-vivo* SPECT/CT imaging and BSGI were selected for histological evaluation. We also selected four aortas of ApoE^−/−^ mice, treatment mice and three control mice without imaging for the same evaluation (Fig. 3). Briefly, all aortas were fixed in formalin, dehydrated, embedded in paraffin and sliced in the region of the aortic arch, thoracic aorta and abdominal aorta that presented with obvious plaque. Serial sections (5 μm thick) were prepared for Hematoxylin-Eosin (HE) and Masson trichrome staining.

Additionally, CD31 and VEGF immunohistochemical staining were also performed on the consecutively cut specimens as follows: serial sections (5 μm thick) were deparaffinized and rehydrated through a series of xylenes and graded alcohols before undergoing antigen retrieval pretreatment (sodium citrate-hydrochloric acid buffer solution for 30 min). Samples were treated with 0.3% H_2_O_2_ for 30 min, followed by blocking with Fatal Bovine serum (Gibco, USA) for 1 hour to prevent nonspecific binding. The sections were then incubated overnight at 4 °C with a primary antibody (anti-CD31 antibody or anti-VEGF antibody, 1:100 in blocking serum; Abcam). A secondary antibody was then applied in GTVision^TM^ III Detection System/Mo&Rb (Gene Tech Company Limited, shanghai, China). Digital images of the stained sections were obtained with a scanning light microscope (Leica Microsystems; Germany).

### Statistical analysis

All statistical analyses were performed with SPSS19.0 (IBM, Chicago, IL, USA) and GraphPad Prism 5.01 (GraphPad Software Inc.) software. Results were expressed as mean ± SD. Unpaired Student’s *t* tests were performed to evaluate the significance of differences in body weight, the weight of abdominal fat, plasma lipid and inflammatory factor levels, as well as plaque uptake of ^99m^Tc-MAG_3_-bevacizumab among different groups. A two-tailed with a *P*-value < 0.05 was considered statistically significance.
